# Adsorption of CO_2_, CH_4_ and H_2_ onto zeolite 13 X: Kinetic and equilibrium studies

**DOI:** 10.1016/j.heliyon.2024.e40672

**Published:** 2024-11-28

**Authors:** Hedi Jedli, Souhail Mohammed Bouzgarrou, Rym Hassani, Ehab Sabi, Khalifa Slimi

**Affiliations:** a University of Monastir, National Engineering School of Monastir, Laboratory of Studies of Thermal Systems and Energy, LR99ES31, 5019, Monastir, Tunisia; b Civil and Architectural Engineering Department, College of Engineering and Computer Sciences, Jazan University, Jazan, Saudi Arabia; c Higher Institute of Applied Sciences and Technologie of Sousse, Sousse University, Tunisia; d Chemistry Department, Center for Environmental and Nature Research, Jazan University, P.O Box 114, Jazan, Saudi Arabia

**Keywords:** Zeolite 13 X, Kinetic, Langmuir, Heat of adsorption

## Abstract

Adsorption equilibrium and kinetics of CO_2_, H_2_ and CH_4_ on zeolite 13 X were carried out using the volumetric method. The zeolite 13 X was characterized via XRD, SEM and N_2_ adsorption-desorption. Adsorption isotherms of various gas were investigated over a full range of temperatures at 298, 308 and 313 K. Langmuir's equilibrium model and the heat of adsorption was used to correlate the adsorption isotherms over a wide range of pressure. A classical micropore diffusion approach was applied to investigate the kinetic adsorption. At 298 K, the selectivity of CO_2_/H_2_ and CO_2_/CH_4_ of the zeolite 13 X was 16.52 and 62.61, respectively. However, zeolite 13 X is one of the most suitable adsorbents for CO_2_ and CH_4_ storage, since it has a very high adsorption capacity for both gases. The influence of gas pressure on diffusivity for various absorber-adsorbent systems has been examined. The adsorption experiments with gas mixtures such as H_2_, CO_2_ and CH_4_ on 13 X zeolite show that this adsorbent has potential application in the gas separation and purification process.

## 1 Introduction

Carbon dioxide is considered one of the greenhouse gases that are responsible for climate change, and is found in many resources such as natural gas, biogas, flue gas [1]. Several studies have been performed on the CO_2_ capture from flue gases related to the combustion and the chemical operations from stationary point sources [2]. A variety of separation techniques have been designed to extract the CO_2_ and to produce useful the constituents from the various gases [3].The adsorption process is the most widely utilized technology for the purification and removal of different gaseous effluents in many industrial applications [[4], [5]]. Several adsorption techniques have been developed to CO_2_ capture from electric stations, including N_2_ and CO_2_ [6]. In addition, the adsorption technologies used in purification and separation are necessary for the CH_4_ production from the coal beds. In fact, CH_4_ can be generated by storage of CO_2_ in coal reservoirs, and can be extracted from exhaust emissions [[7], [8]]. The use of natural gas as a fuel source is increasing sharply, and the valorization of these gases is therefore being studied in terms of adsorption, since this gas is composed of a mixture of CO_2_ and N_2_ [9]. In addition, the growing demand for hydrogen has led to the processes development for the production of high-purity hydrogen from synthesis gas, coke oven gas and refinery fuel gas. Adsorption processes based on solid sorbents such as TSA (Temperature Swing Adsorption) and PSA (Pressure Swing Adsorption) is widely employed to CO_2_ extraction and to generate clean energy, such as CH_4_ and H_2_, from different waste gases [10,11]. This process has been used to separate and purify various gas mixtures [12]. Different emitted gases, such a refinery gas, combustion gas, coal bed methane and coal synthesis, are composed of a mixture of carbon dioxide, methane and dihydrogen following pretreatment by removal of acid gases and water [13]. Since various effluent gases have high component content, the study of kinetics adsorption equilibrium and heats of adsorption of the component on the adsorbent are necessary for the adsorption processes. Numerous studies have been done on the adsorption equilibrium and kinetics of zeolite sample for some gas effluent components.

The adsorption performance on zeolites at various pressures has been investigated for CO_2_, CO, CH_4_, N_2_, and H_2_ on zeolite 5 A and SSZ-13 zeolite [14,15], N_2_, CO_2_ and CH_4_ on zeolite 13 X [16,17], and CO_2_, CO, N_2_, CH_4_ and H_2_ on zeolite LiX [18] by means of various experimental approaches. In additional, the binary mixing of adsorption equilibria has been investigated on various zeolites [19,20]. The CO_2_ diffusion mechanism is an essential parameter for understanding the adsorption process and has been cited in many works such as X Hu et al. (2014) [21] and MI Hossain et al. (2019) [22]. Among the different zeolites utilized in separation technology, zeolite 13 X is currently being applied for the development of gas extraction systems [16,[23], [24], [25]].

The aim of this paper is to study the zeolite 13 X as solid adsorbent for CO_2_, CH_4_ and H_2_ adsorption. The adsorption isotherm was measured at three temperatures (293, 308 and 323 K) and modeled with the Langmuir and Freundlich model. The adsorption kinetics and the heat adsorption for CO_2,_ H_2_ and CH_4_ were calculated and analyzed. The results obtained show that zeolite 13 X can contribute to the adsorption of gases including H_2_, CH_4_ and CO_2_.

## 2 Experimental section

### 2.1 Materials

Powder zeolite 13 X, was taken from Sigma-Aldrich. High purity gases, i.e. CO_2_ 99.995 %, CH_4_ 99.999 % and H_2_ 99.995 % utilized in the measurements were obtained by Linde Sdn Bhd.

### 2.2 Characterizations

The X-ray diffraction patterns of the zeolite were obtained using a diffractomètre Panalytical Xpert Pro in the 2 Theta with Cu k α. The zeolite used in the same purchased state, has been characterized with Thermo Fisher FEI Q250 scanning electron microscope with an acceleration voltage of 10–15 kV. The textural properties of the material were determined through physical adsorption/desorption of N_2_ at −196 ^°^C on a Micromeritics ASAP 2010 device. The Brunnauer-Emmet-Teller (BET) equation was applied to estimate the surface area. The total pore volume (Vp) was calculated from the adsorbed volume of nitrogen for a relative pressure (p/p_0_) of 0.97. The micropore volume (V_micr_) was calculated by applying the Dubinin-Radushkevich method at p/p_0_ < 0.1, and the mesopore volume (V_mes_) was estimated as V_mes_ = V_T_ – V_micr_ [26].

### 2.3 Adsorption experiment

The adsorption isotherms on all 13 X were measured using a Micromeritics ASAP 2020 analysis system at 298, 308 and 313 K. The sample (0.08 g) was was degassed under vacuum at 250 °C for more than 12 h to remove adsorbed contaminants prior to the measurement. The sample was then subjected to gas adsorption (CO_2_, CH_4_ and H_2_) at a temperature of 0 °C in a bath with controlled water circulation. A target pressure was gradually raised to around 800 mmHg, and at each target pressure point, the quantity of adsorbed was recorded. For desorption, the pressure in the sample tube is gradually reduced by vacuum, and the volume of CO_2_ gas is established at each pressure point [27].

After measuring the adsorption capacity, the equilibrium selectivity of the various gas was determined using the ratio of Henry's law constants (equation (1) [28]:

(1) $$S_{1,2}=\frac{K_{1}}{K_{2}}$$

With K_1,2_ being the Henry's law constant of the elements 1 and 2 respectively. The Henry constants were derived by calculation of the initial isotherms slope.

## 3 Adsorption models

### 3.1 Isotherm models

In this study, the CO_2_, CH_4_ and H_2_ adsorption on the zeolite 13 X was adjusted using the Langmuir and Freundlich model. We note that Langmuir model was applied to describe monolayer adsorption onto the homogeneous surface. It is expressed by the following equation (2) [29]:

(2) $$q=q_{m}\left[kp/\left(1+kp\right)\right]$$

Where *q* and $q_{m}$ represent respectively the equilibrium adsorption capacity and the saturation capacity of the gas adsorbed. *k* was the Langmuir constant and *p* was the equilibrium pressure.

On the other hand, Freundlich isotherm model is applicable to the multilayer adsorption, and is expressed as follows [29]:

(3) $$q=k_{F}\;P^{1/n}$$

Where $k_{F}$ is the Freundlich isotherm constant and *n* is the heterogeneity factor.

The validity of this model was assessed by the standardized standard deviation Δq(%), which is expressed as follows [29]:

(4) $$\Delta q\left(\%\right)=100\sqrt{\frac{\sum {\left[\left(q_{\exp}-q_{\mathrm{mod}}\right)/q_{\exp}\right]}^{2}}{N-1}}$$

Where N, $q_{\mathrm{mod}}$ and $q_{\exp}$ represent respectively the number of adsorption isotherm data points, the adsorption capacities obtained from the model and the adsorption capacities obtained from the experiments.

### 3.2 Isosteric heat of adsorption

The details related to the heat release during adsorption are essential for understanding the affinities and adsorption kinetics. Part of the adsorbed heat rises the temperature of the particles, and this increase in the temperature influences on the separation performance and on the adsorption kinetics [30].

The isotropic heat of adsorption ($Q_{st}$) is an essential factor to characterize the interaction force by the adsorbent surface and the adsorbed molecules. $Q_{st}\left(\frac{kj}{mol}\right)$ was obtained from the experimental isotherms at various temperatures. In this study, the isosteric heat of adsorption was obtained using the Clausius-Claypeyron equation and the Langmuir model [9,30]:

(5) $$\frac{Q_{st}}{R}={\left(\frac{dlnp}{d\left(\frac{1}{T}\right)}\right)}_{q}$$

Where *p* and *T* represent the equilibrium pressure and the temperature. The isosteric heats of adsorption were obtained by plotting the values of ln *p* versus of the *1/T*.

### 3.3 Adsorption kinetics

The adsorption kinetics of CO_2_, CH_4_ and H_2_ on the mesoporous 13 X were measured at 298 K and a low pressure (2 kPa). The diffusion time constants can be obtained by adjusting the fractional absorption plot with an appropriate diffusion model and is expressed by the following equation [31,32]:

(6) $$1-\frac{m_{t}}{m_{\infty }}=\frac{6}{\pi^{2}}\exp\left(\frac{-\pi^{2}D_{c}t}{r_{c}^{2}}\right)$$

Where $\left(\frac{m_{t}}{m_{\infty }}\right)$ the fractional adsorption, and $\left(\frac{D_{c}}{r_{c}^{2}}\right)$ is the diffusion time constant. $m_{t}$ and $m_{\infty }$ represent respectively the quantity of gas adsorbed at time t and the total quantity of gas adsorbed at equilibrium pressure. $D_{c}$ is the intracrystalline diffusivity and $r_{c}$ was the sphere radius.

Diffusion time constants ($\frac{D_{c}}{r_{c}^{2}}$, s^−1^) were determined from the slope of a linear plot of ln(1-($\frac{m_{t}}{m_{\infty }}$)) versus t (time) at a constant pressure. To estimate the diffusion time constants, the data points with ($\frac{m_{t}}{m_{\infty }}$) higher than 70 % and lower than 99 % were used. The intracrystalline diffusivity ($D_{c}$) of the various gases was determined by multiplication of the diffusion time constant by $r_{c}^{2}$ values.

## 4 Results and discussion

### 4.1 Structure characterization

**Fig. 1:**
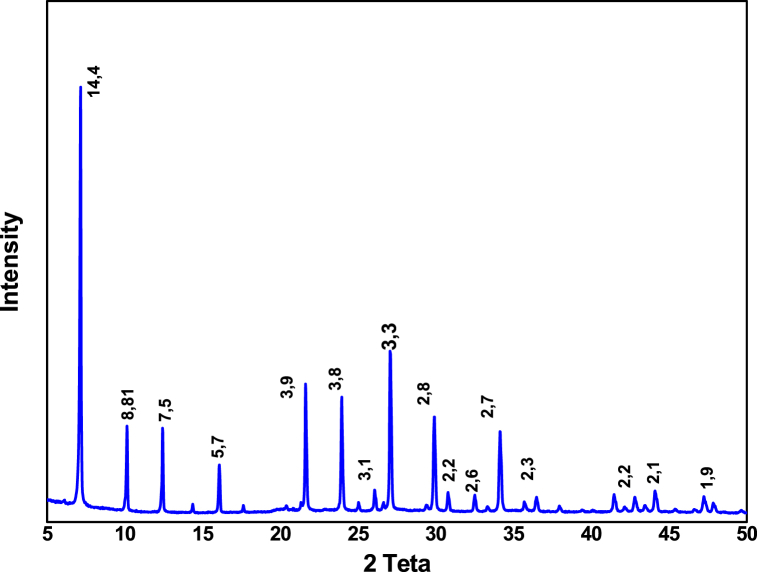
XRD of zeolite 13 X.

**Fig. 2:**
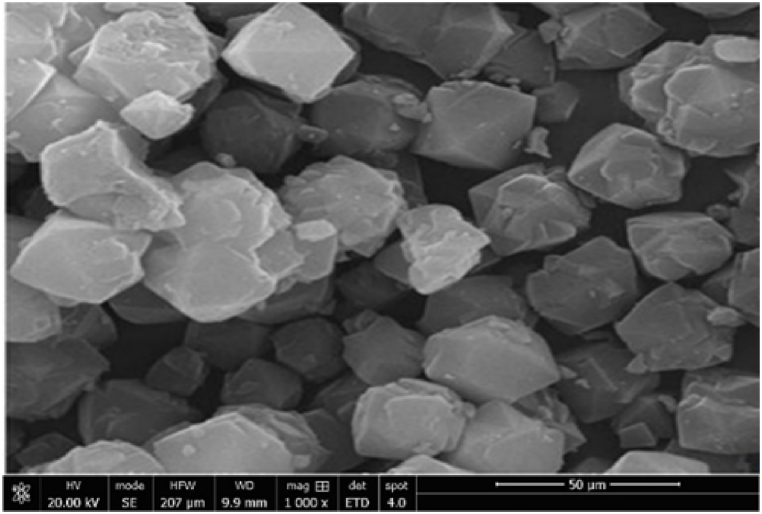
SEM of zeolite 13 X.

**Fig. 3:**
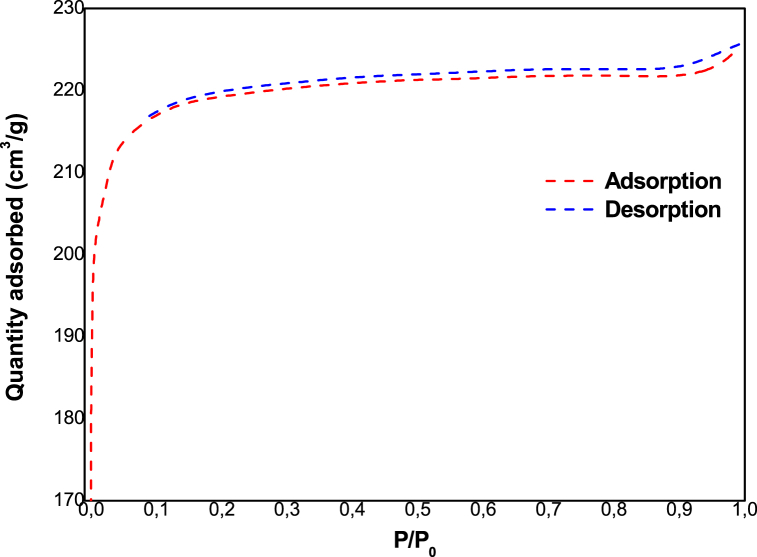
N_2_ adsorption – desorption of zeolite 13 X.

Fig. 1 shows the XRD of zeolite 13 X, which shows the presence of the main peaks at 2θ = 6.1°, 10°, 15.5°, 20.1°, 23.3°, 26.7°, 29.3°, 30.5°, 31.0° and 32.1°. Fig. 2 shows the SEM micrograph of zeolite 13 X. We can see that the structure of this zeolite is composed of octahedral crystals. Moreover, these crystals are formed by non-uniform particles with irregular surfaces. Fig. 3 shows that the nitrogen adsorption-desorption isotherms of zeolite 13 X are similar type IV adsorption isotherms to typical mesoporous materials with H3 type hysteresis [31]. We can see that the adsorption process is accelerated at low pressure because the adsorption sites are empty, then a plateau is reached when the micropores are saturated with adsorbates. At high relative pressure, the low desorption hysteresis loop is attributed to mesopore filling. Textural parameters including such as the BET surface area and the total volume are given in Table 1.

**Table 1:** Textural parameters of zeolite 13 X.

Adsorbent	BET surface area (m^2^/g)	Vtotal (cm^3^/g)	Vmesopore (cm^3^/g)	Vmicropore (cm^3^/g)
13 X	786	0.37	0,22	0.15

### 4.2 Adsorption isotherms

**Fig. 4:**
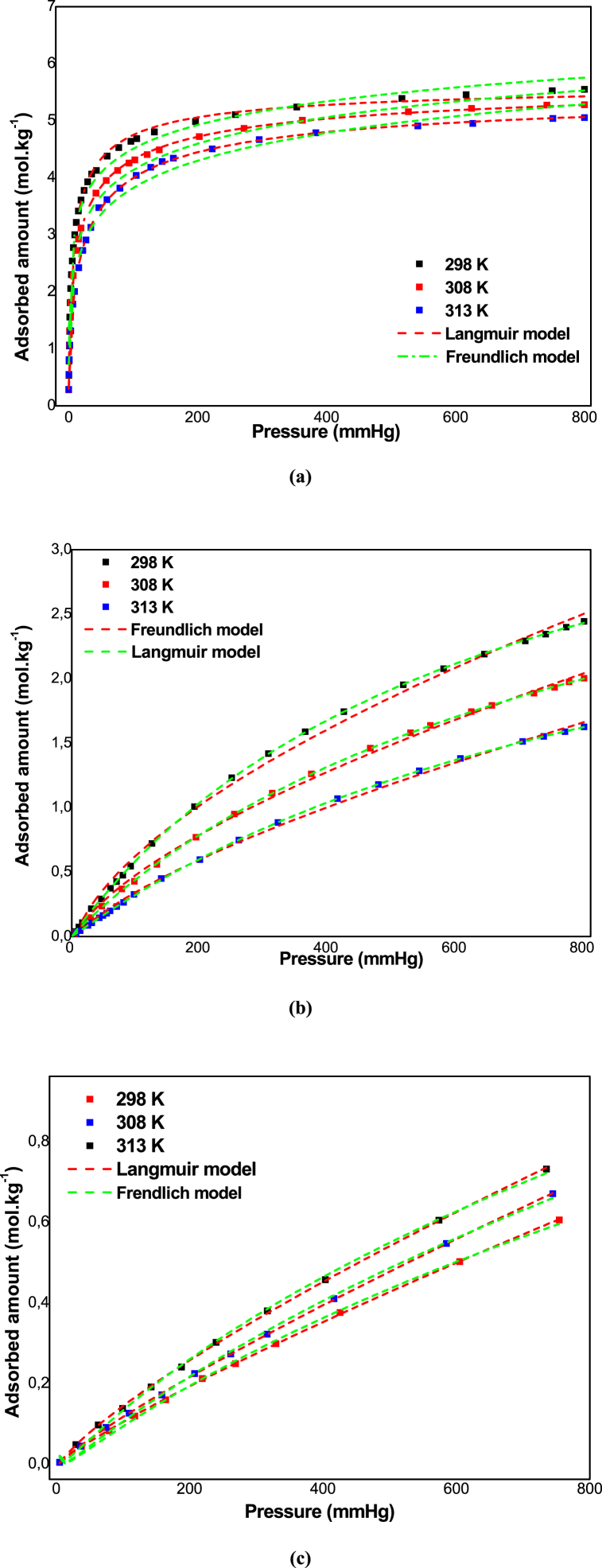
Adsorption isotherms of the (a) CO_2_, (b) CH_4_ and (c) H_2_ on the zeolite 13 X.

The adsorption isotherms for CO_2_, CH_4_ and H_2_ obtained at different temperatures (298, 308 and 313 K) are reported in Fig. 4(a) and (b) and (c), respectively. We can note that the adsorption capacity rises with the increase in the pressure of the gases studied. On the other hand, an increase in adsorption temperature means a decrease in the quantity of the gas adsorbed, which indicates that the adsorption process is exothermic [23].

According to Fig. 4, the CO_2_ and CH_4_ adsorption isotherms on zeolite 13 X present a convex type I curvature, whereas the H_2_ isotherm were practically linear. Moreover, CO_2_ is the most adsorbed gas, with a quantity of 3.3 mol kg^−1^ at 303 K and 800 mmHg. At the same pressure and temperature, the CH_4_ and H_2_ amount adsorbed is much lower, at around 2.2 and 0.78 respectively. As a result, the degree of adsorption was CO_2_> CH_4_>H_2_ for the same pressure and temperature range.

The experimental adsorption was fitted with Langmuir and Freundlich model. The marker points depict the experimental data and the solid lines show the isotherm model applied in the present work. Table 2 gives the model parameters including the correlation coefficient (R2) and Δq(%). Following Fig. 4, the both model showed good agreement with the experimental adsorption isotherms. This demonstrated by the fact that R2 is higher than 0.98. According to the Langmuir parameters summarized in Table 2, the values of *q*_*s*_ and *k* reduce with increasing in the temperature, suggesting the exothermic adsorption process [29].

**Table 2:** Langmuir parameters of zeolite 13 X.

Gas	Temperature (K)	Langmuir model					Freundlich model				
		q_m_ (mol.kg^−1^)	k (mmHg^−1^)	R	Δq(%)	Δk(%)	q_m_ (mol.kg^−1^)	$k_{F}$ (mmHg^−1^)	R	n	Δq(%)
CO_2_	298	5.332	6.79	0.9968	1.85	2.31	5.215	5.21	0.9218	3.21	1.78
	308	5.142	6.42	0.9997	1.93	2.41	5.102	5.01	0.9101	3.15	1.89
	313	4.962	6.01	0.9998	2.31	2.32	5.013	4.98	0.9048	3.02	2.28
CH_4_	298	4.419	2.41	0.9978	1.81	1.85	4.315	2.11	0.9974	2.98	1.80
	308	4.070	2.38	0.9996	1.86	1.92	4.121	1.98	0.9981	2.91	1.82
	313	3.714	2.21	0.9995	1.92	1.98	4.015	1.78	0.9978	2.88	1.90
H_2_	298	4.102	1.79	0.9988	1.49	0.67	4.091	1.42	0.9972	2.77	1.42
	308	3.982	1.42	0.9996	1.72	0.97	3.887	1.38	0.9996	2.45	1.69
	313	3.978	1.01	0.9994	2.01	1.21	3.789	1.29	0.9984	2.33	1.98

From Tables 1 and it's clear that the Freundlich equation seems to fit the data better over the partial pressure range, which indicates the heterogeneous surface. The parameter n is always greater than 1, confirming that the adsorption process is a physisorption approach. Moreover, 1/n values are below 1 for all temperatures, which suggests excellent adsorption intensity [30]. Nevertheless, the decrease in k_F_ and n parameters with the increase in temperature indicates that adsorption is favorable at low temperatures and is therefore an exothermic adsorption phenomenon.

The gas selectivity obtained from the isotherms are listed in Table 3. We note that the obtained selectivity are applicable at very low pressures, owing to the limitations of Henry's law. It can be noted that the CO_2_/CH_4_ equilibrium selectivity is higher than that of CO_2_/H_2_, which is 52.61 and 16.52 respectively. In addition, the CO_2_/CH_4_ and CO_2_/H_2_ selectivity decreases with increasing temperature. In addition, in the same table, we can see that the difference between the diffusion time constants of CO_2_, H_2_ and CH_4_ is small, which means that adsorptive separation is efficient. Furthermore, for each gas the diffusion time constant rises slightly with the increase in temperature.

**Table 3:** Equilibrium selectivity and diffusion time constants of CO_2,_ CH_4_ and H_2_ on zeolite 13 X at different temperatures.

	298 K	308 K	313 K
CO_2_/H_2_	16.52	16.03	11.09
CO_2_/CH_4_	52.61	51.13	42.78
D_c_/r^2^_c_ (10^−2^s^−1^) CO_2_	1.67	2.08	2.87
D_c_/r^2^_c_ (10^−2^s^−1^) CH_4_	3.6	4.61	5.21
D_c_/r^2^_c_ (10^−2^s^−1^) H_2_	3.20	4.110	4.67

### 4.3 Adsorption kinetics

**Fig. 5:**
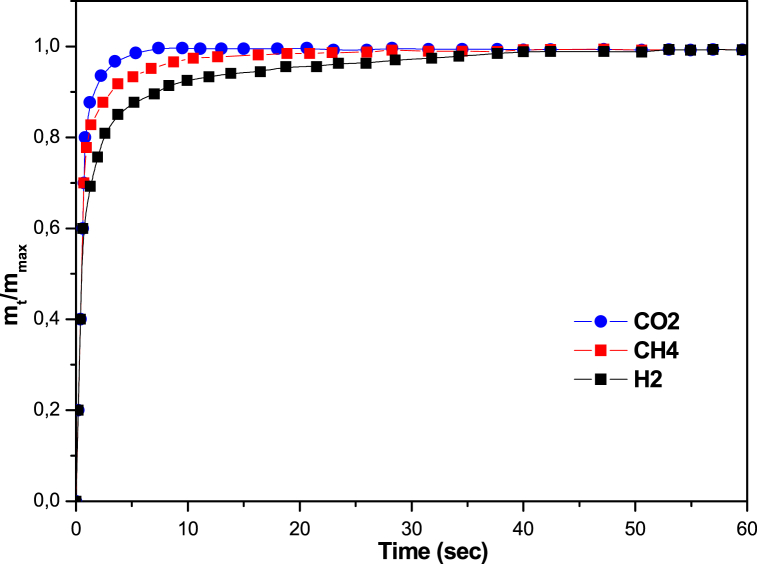
Adsorption kinetics of the CO_2_, CH_4_ and H_2_ on zeolite 13 X.

The kinetic adsorption curves for CO_2_, H_2_ and CH_4_ on the adsorbent are shown in Fig. 5. Kinetic curves for various gases of similar shape. We can see that the diffusivity of CO_2_ is higher than that of CH_4_ and H_2_ in zeolite 13 X. The figure shows that 13 X reached adsorption saturation within a time interval of between 30 and 60 s. The radius $r_{c}$ of the 13 X zeolite was assessed from their SEM images represented in Fig. 2, was 0.46 × 10^−6^ m.

**Fig. 6:**
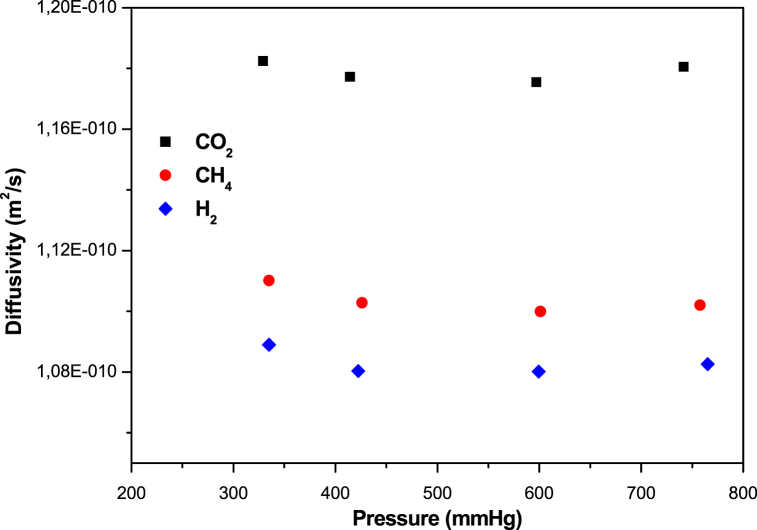
Variation of the CO_2_, CH_4_ and H_2_ diffusivity in zeolite 13 X.

The variation in diffusivity with the variation in pressure is plotted in Fig. 6 for CO_2_, CH_4_ and H_2_ on zeolite 13 X. From the graphs, it's clear that the magnitude of diffusivity reveals a declining trend with increased of the pressure. The decreasing character of diffusivity can be ascribed to the nature of the adsorbent pores, which are partially saturated and blocked at high adsorption loads under higher pressures, resulting in slow kinetics. In the example of CO_2_ adsorption on zeolite 13 X, the high adsorption charge of the molecule adsorbed on the material means a higher surface concentration, causing the molecules to slide across the surfaces. This slippage leads to surface diffusion at the highest adsorption load at the greatest pressure, and this surface diffusion increases overall intracrystalline diffusivity with rising pressure [21].

### 4.4 Isosteric heat of adsorption

**Fig. 7:**
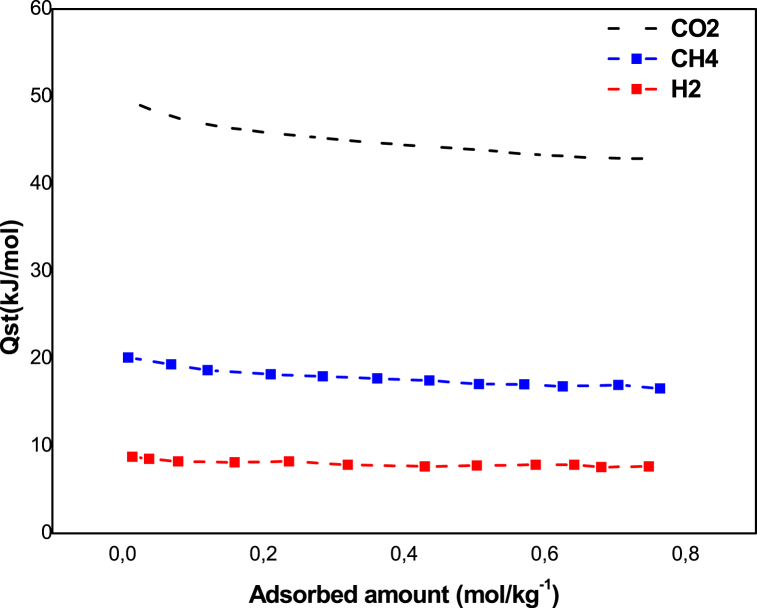
Isosteric heat of adsorption zeolite 13 X.

The isosteric heat was obtained by the Clausius-Claypeyron equation and with the Langmuir model. Fig. 7 shows that the isosteric heat of CO_2_ is greater than that of CH_4_ and H_2_ in the low and high surface load ranges. We can note that the adsorption isosteric is independently of the quantities adsorbed when the surface is energetically homogeneous without interaction between the adsorbed molecules [9]. We can note that the interaction between the adsorbate and the adsorbent (vertical interaction) initially takes place at a lower surface charge and leads in a raised heat of adsorption [9]. In addition, the lateral interaction is observed with the rising on the surface charge [9]. Consequently, the heat of adsorption variation with the surface charge can be ascribed to lateral interaction between adsorbed gases with the exception of H_2_. Therefore, the adsorption of CO_2_ and CH_4_ gas involves a vertical interaction resulting from the energetic heterogeneity surface.

The development of an efficient material for CO_2_ adsorption is important to develop energy-efficient solutions for a green environment. The CO_2_ absorption amount is one of the performance criteria for solid adsorbents. Table 4 present the CO_2_ adsorption capacity of zeolite 13 X compared with various zeolites published in the literature. The comparison between 13 X zeolite and a number of adsorbents reveals that 13X has a comparable or higher adsorption capacity than other types of adsorbent, such as Na-Zeolite β and Zeolite 13 X (13 X -F). Therefore, the utilization of 13 X as an adsorbent in the separation processes presents a greater potential owing to its high porosity and adsorption capacity.

**Table 4:** Comparison of the CO_2_ adsorption capacities by different adsorbents.

Adsorbent	Temperature (K)	CO_2_ uptake (mmol/g)	Reference
Zeolite 13 X (13 X -B)	298	4.8	[33]
Zeolite 13 X	298	4.7	[34]
Zeolite 13 X	298	3.2	[35]
Na-Zeolite β	298	2.8	[36]
Zeolite 13 X (13 X -F)	298	3.9	[37]
Zeolite 13 X (13 X -B)	298	4.2	[37]
Zeolite 13 X	298	5.3	This work

## 5 Conclusion

The adsorption isotherms of CO_2_, CH_4_ and H_2_ on zeolite 13 X were measured at 293, 308 and 318 K and at pressure up to 800 mmHg. The zeolite 13 X was characterized via XRD, SEM and N_2_ adsorption - desorption. Langmuir and Freundlich isotherm models was performed to correlate adsorption isotherms. The order of adsorption and the heat of adsorption was: CO_2_ >CH_4_>H_2_. The CO_2_ and CH_4_ presented a strong vertical interactions; however, lateral interactions improved with increasing load. Although the adsorbed amount of H_2_ were lateral interactions. A diffusion model was applied to evaluate the adsorption kinetics and the influence of gas pressure on diffusivity for different absorber-adsorbent systems was examined. The zeolite 13 X present an excellent performance for the separation of CO_2_/H_2_ and CO_2_/CH_4_.The equilibrium selectivity of CO_2_/H_2_ and CO_2_/CH_4_ were 16.52 and 62.61 at 298 K respectively. The adsorption experiments demonstrated that the zeolite 13 X have a potential application to separation and gas purification process.

## CRediT authorship contribution statement

**Hedi Jedli:** Conceptualization. **Souhail Mohammed Bouzgarrou:** Data curation. **Rym Hassani:** Conceptualization. **Ehab Sabi:** Data curation. **Khalifa Slimi:** Conceptualization.

## Data availability statement

Data will be made available on request.

## Declaration of competing interest

The authors declare that they have no known competing financial interests or personal relationships that could have appeared to influence the work reported in this paper.
